# Comparing genome-scale DNA methylation and CNV marks between adult human cultured ITGA6+ testicular cells and seminomas to assess in vitro genomic stability

**DOI:** 10.1371/journal.pone.0230253

**Published:** 2020-03-16

**Authors:** Robert B. Struijk, Lambert C. J. Dorssers, Peter Henneman, Martin A. Rijlaarsdam, Andrea Venema, Aldo Jongejan, Marcel M. A. M. Mannens, Leendert H. J. Looijenga, Sjoerd Repping, Ans M. M. van Pelt

**Affiliations:** 1 Center for Reproductive Medicine, Research Institute Reproduction and Development, Amsterdam University Medical Center, University of Amsterdam, Amsterdam, The Netherlands; 2 Department of Pathology, Erasmus MC University Medical Center, Rotterdam, and Princess Maxima Center for Pediatric Oncology, Utrecht, The Netherlands; 3 Department of Clinical Genetics, Amsterdam University Medical Center, University of Amsterdam, Amsterdam, The Netherlands; 4 Bioinformatics Laboratory, Department of Clinical Epidemiology, Amsterdam University Medical Center, University of Amsterdam, Amsterdam, The Netherlands; Massachusetts General Hospital, UNITED STATES

## Abstract

Autologous transplantation of spermatogonial stem cells is a promising new avenue to restore fertility in infertile recipients. Expansion of the initial spermatogonial stem cell pool through cell culturing is a necessary step to obtain enough cells for effective repopulation of the testis after transplantation. Since *in vitro* propagation can lead to (epi-)genetic mutations and possibly malignant transformation of the starting cell population, we set out to investigate genome-wide DNA methylation status in uncultured and cultured primary testicular ITGA6+ sorted cells and compare them with germ cell tumor samples of the seminoma subtype. Seminomas displayed a severely global hypomethylated profile, including loss of genomic imprinting, which we did not detect in cultured primary testicular ITGA6+ cells. Differential methylation analysis revealed altered regulation of gamete formation and meiotic processes in cultured primary testicular ITGA6+ cells but not in seminomas. The pivotal *POU5F1* marker was hypomethylated in seminomas but not in uncultured or cultured primary testicular ITGA6+ cells, which is reflected in the *POU5F1* mRNA expression levels. Lastly, seminomas displayed a number of characteristic copy number variations that were not detectable in primary testicular ITGA6+ cells, either before or after culture. Together, the data show a distinct DNA methylation patterns in cultured primary testicular ITGA6+ cells that does not resemble the pattern found in seminomas, but also highlight the need for more sensitive methods to fully exclude the presence of malignant cells after culture and to further study the epigenetic events that take place during *in vitro* culture.

## Introduction

*In vitro* propagation of cryopreserved spermatogonial stem cells (SSCs) followed by autologous transplantation of cultured SSCs (SSCT) into the testes is viewed as a promising new technique to treat male survivors of childhood cancer for sub- or infertility [1–5]. Theoretically, by utilizing SSCT in this group of otherwise infertile patients, endogenous spermatogenesis can be permanently enhanced or rescued. SSCT has the additional benefit of using the patient’s own cells to rescue fertility, rendering SSCT a preferred option to current clinical alternatives such as the use of donor sperm to achieve pregnancy. The robustness of spermatogenic rescue following SSCT has been demonstrated for various species including mice, cattle and primates [6–10] and SSCT treated animals are capable of producing offspring which appears healthy [11–15] and fertile, at least in rodents [12, 14–16]. The odds of successful testicular colonization after SSCT are predominantly dictated by the number of transplanted SSCs [17]. Since the proportion of true SSCs will be limited in a biopsy from a human prepubertal small testis, *in vitro* propagation of the initial SSC pool is a necessary step in the SSCT protocol.

A potential risk with SSCT for clinical use is the risk of cancer induction in the recipient originating from the transplanted cell population [18, 19]. This risk is two-fold: either primary cancerous cells (originating from non-solid tumors) that were present in the original biopsy can be re-introduced into the recipient upon transplantation, or normal germ/somatic cells could give rise to a transformed line of cancer cells with malignant properties during *in vitro* propagation. Several studies have been published that describe the use of different techniques, such as cell culture or FACS, to successfully eliminate malignant cells from contaminated testicular tissue samples [20–22]. The possible secondary risk of testicular cells undergoing culture-induced malignant transformation remains largely unexplored in the context of fertility restoration.

A common hallmark of malignant cells is the occurrence of disturbances in 5-cytosine methylation marks, resulting in an epigenetic landscape that may differ greatly from normal cells [23]. Such alterations in DNA methylation have been found in testicular malignant germ cell tumors of all histological variants, including seminomas, as well its precursor lesion germ cell neoplasia *in situ* (GCNIS, previously known as CIS) [24–26]. In addition to alterations in their methylome, these malignant cells are often susceptible to and characterized by DNA copy number variations and can in fact even be subclassified based on the occurrence of CNVs in certain loci [27], including TGCTs [28].

Due to the available evidence that *in vitro* proliferation of primary (stem) cells can affect both the genetic [29, 30] and epigenetic [31–34] integrity of the cell’s genome and possibly trigger malignant transformation, we set out to study whether primary human testicular cells can become malignant during propagation in cell culture. Sporadic genetic aberrations have been observed in long-term cultures of induced pluripotent stem cells (iPS) and embryonic stem cells (ES) [35, 36], displaying recurrent duplications of chromosomal regions associated with increased genetic instability and apoptotic resistance (12q, 17p) also found in various GCNIS-derived TGCTs.

Here, we investigated genome-wide methylation patterns and genomic CNVs in freshly sorted and long-term cultured and sorted integrin alpha-6 (ITGA6) positive human testicular cells (n = 4 cultures) and compared these methylation patterns to primary seminoma tumors (n = 3), focusing on genome-scale DNA patterning as well as known oncogenic regions. Selection of ITGA6 positive cells was based on its localization to the plasma membrane of spermatogonia in the *in vivo* human testis [37] and previous demonstrations of the testis-repopulating capacity of the ITGA6+ testicular cell subpopulation, both before and after culturing [38–41].

## Materials and methods

### Ethical approval

This study does not report research involving human participants. We used spare human testicular tissue fragments with written or oral informed consent from prostate cancer patients (n = 4) ranging in age from 49–83, that underwent bilateral orchidectomy as part of their cancer treatment at the Academic Medical Center (AMC), Amsterdam, The Netherlands. In accordance with Dutch law, spare tissues can be used for research with permission of the patients without further permission of an ethical committee since no additional interventions were needed to obtain these samples. Seminoma DNA samples, also used in a previous study [25], were obtained with informed consent from patients in the Netherlands and were handled according to the Code for Proper Secondary Use of Human Tissue as directed by the Dutch Federation of Medical Scientific Societies (https://www.federa.org/codes-conduct).

### Patient samples

Adult testicular donor tissues were cryopreserved in 1× MEM, 20% FCS, 8% DMSO upon retrieval and stored for later cell isolations. Haematoxylin-eosin stainings of testicular cross sections were used to confirm the presence of all germ cell stages and revealed normal spermatogenesis in all donor tissues. Seminoma samples were stage I tumors that did not show metastases during follow-up. Seminoma samples were obtained and used directly after surgery. Part of the biopsies were submitted to the pathology department for histological examination and alkaline phosphatase (dAP) reactivity assays [42], the remainder was subjected to DNA isolation as described in previous work [25]. Lymphocyte infiltration scoring showed moderate to high infiltration in two of the tumors (L11-119: 57.5%-66.1% and L11-123: 68.4%-71.1%) and moderate infiltration in the remaining tumor (L10-358: 37.3%-52.5%) with an expected overestimation of 10–20% lymphocyte component.

### Primary testicular cell isolation and culture

Biopsies were thawed and the germ cell fraction located on the basal membrane of seminiferous tubules was isolated as described previously [43]. Briefly, testicular biopsies were subjected to a two-step enzyme digestion (1mg/mL type I collagenase (4196, Worthington), 1mg/mL type II hyaluronidase (H2126, Sigma) and 1mg/mL trypsin TRL3 (3707, Worthington), followed by 1mg/mL type I collagenase and 1mg/mL type II hyaluronidase) and the resulting single-cell suspension was plated overnight in 1× MEM containing 20% FCS. The next day, floating cells (containing the germ cell fraction) were separated from the attached cell fraction via gentle pipetting and transferred to a new culture flask. This mixed primary testicular cell (PTC) fraction was then either directly subjected to magnetic-activated cell sorting for ITGA6+ cells (day 0; d0-PTC) to enrich for spermatogonia or first brought into culture and then sorted (long-term; LT-PTC). The medium used for PTC culture contained StemPro-34 SFM medium (10639–011, Life Technologies) supplemented with several key growth factors promoting spermatogonial stem cells self-renewal (GDNF, EGF, PDGF, LIF) as described elsewhere [43]. Cells were cultured at 37°C and 5% CO_2_ and passaged using trypsin/EDTA at 80–90% confluence, which took on average 13 days for the first passage and from that point on every 7 to 10 days over a period of 50–54 days. Medium was refreshed twice a week.

### Magnetic-activated cell sorting

To enrich the initial testicular cell fractions for spermatogonia before and after culture, we performed magnetic assistant cell sorting (MACS) for cells expressing the ITGA6 membrane protein [38–40]. Cells were harvested using 0.25% trypsin/EDTA, filtered using a 40 μm sterile filter and labeled with biotinylated anti-ITGA6 antibody (BioLegend, 1μl ab per 5 × 10^5^ cells) in 100 μl MACS buffer (0.5%BSA, 2mM EDTA in sterile PBS) for 30 minutes at 4°C, followed by incubation with 1:5 (v:v) anti-biotin micro magnetic beads (Miltenyi Biotec) in 100 μl MACS buffer for 15 minutes at 4°C. Cell-bead complexes were then washed 3× in MACS buffer and transferred to a magnetic stand to separate labeled cells from non-labelled cells using a Large Cell separation column (Miltenyi Biotec).

### DNA isolation and bisulfite conversion

Genomic DNA was isolated from ITGA6+ PTCs using the QIAamp DNA Mini Kit (Qiagen, 51306) and eluted in low-TE buffer (10 mM Tris-HCl, 0.1 mM EDTA in sterile H_2_0, pH 8.0). Concentration and quality of DNA was measured on a ND-1000 (Thermo Scientific) spectrophotometer and 1 μg of high quality DNA (absorbance 260/230 ≥ 1.8; 260/280 ≥ 2.0) was subjected to bisulfite conversion using the EZ DNA Methylation Gold Kit (Zymo Research). High bisulfite conversion rates were confirmed by single curves using high resolution melt analysis (HRMA) of the *H19* imprinting control region 1 (*H19-IC1*) in sperm control DNA as described elsewhere [44], as well as a two-step verification using internal control probes on the Human Methylation-450k BeadChip microarrays. Primers used for HRMA were as follows: forward ATGTAAGATTTTGGTGGAATAT and reverse ACAAACTCACACATCACAACC.

### Human Methylation-450k BeadChip microarray analysis

Methylation profiles of uncultured and cultured ITGA6+ PTCs were generated using HumanMethylation 450k BeadChip microarrays (Illumina), which cover 485,577 genomic CpG sites and capture ≥ 99% of RefSeq genes. Sample preparation and bisulfite quality checks were conducted at the department of Clinical Genetics at the Academic Medical Center (AMC), Amsterdam, the Netherlands. Bisulfite-converted DNA samples were hybridized to the arrays at Service XS, Leiden, the Netherlands according to manufacturer’s protocol. BeadChip images were scanned using an Illumina iScan array scanner and data was extracted into *GenomeStudio* (v2011.1, Methylation Module v1.9.0) using default analysis settings. Data in this study were further processed using R (v3.1.1, platform: x86_64-w64-mingw32/x64) and Rstudio (v0.98.1091) for Windows 7 (64 bits); packages included *lumi* (v2.20.1), *limma* (3.22.7) and *ggplot2* (v1.0.0). The data set supporting the results of this article is available in the Gene Expression Omnibus repository, accession number GSE72444.

### Data preprocessing

Raw scanner data (.iDat files) were imported into R and preprocessed using *lumi* [45]; preprocessing steps included removal of non-CpG probes and non-CpG SNP probes, CpG probes not passing the fluorescence detection limit (detectable signal in 95% of samples, p ≤ 0.01), CpG probes known to have multiple targets in the genome and CpG probes with SNPs at or within 10bp of a target CpG (allele frequency ≥ 0.05) [46]. Preprocessing with these criteria resulted in 452,354/485,577 probes suitable for downstream analysis. Fluorescence values of preprocessed CpG probes were subjected to optimized color-bias adjustment and quantile normalization [47] followed by BMIQ-based correction for type I and type II probe bias [48]. Data processing yielded both ß values (*i*_n,methylated_ / (*i*_n,unmethylated_ + *i*_n,methylated_)), where *i*_*n*_ is intensity signal of the n^th^ probe and related M-values (log_2_(ß_n_ / 1–ß_n_) where ß_n_ is the ß value of the n^th^ probe) for each probe as methylation estimates. We used an extended annotation file (available in the Gene Expression Omnibus repository, accession number GPL18809) and the VariantAnnotation package for CpG annotation, adding a number of functional genomic classes to the standard annotation manifest provided by Illumina.

### DMR identification

We used DMRforPairs (v1.2.0) [49] to identify CpG dense regions and define DMRs in the dataset using default settings. For statistical analysis of candidate DMRs we opted to use M-values as opposed to ß-values due to the higher rates of true discovery [50]. A genomic interval was classified as a DMR when it contained at least 4 CpGs within a distance of ≤ 200bp from each other and showed a significant difference in median M-value of ≥ 1.4 (Benjamini-Hochberg adjusted p-value ≤ 0.05, Mann Whitney U test) between two samples.

### Copy number variation analysis

Copy number variations were calculated using a previously published approach [51] that we modified to also include CpGs on the X chromosome. CpG probes were divided over 8,949 chromosomal bins and median probe fluorescence was calculated for each bin in a DNA methylation microarray control dataset of 10 female blood samples with stable copy numbers. Based on the fluorescence levels in this control set, a baseline was defined for stable copy numbers of all autosomes (chromosomes 1–22) and chromosome X. Bins where the median probe fluorescence in an experimental sample (PTC, SE) was significantly different from bin fluorescence in the control set were assigned as having a CNV in that sample, designated as +1 for gains and –1 for losses. Cutoff levels for the designation of chromosomal copy number variants provided in **S1 File** were adapted from [51], distinguishing between homozygous deletions (≤–0.96, brown), hemizygous/mosaic deletions (≤–0.24, red), neutral (between 0.12 and –0.24, grey), duplications (≥ +0.12, green) and high-copy gains (≥ +0.72, blue).

### Reverse transcriptase PCR & qPCR

Reverse transcriptase and quantitative polymerase chain reactions (PCR) were carried out using amplified RNA from the same cells that were used for DNA methylation profiling. RNA was isolated using a MagnaPure LC machine (Life Technologies) and amplified using an Ovation RNA-Seq System V2 kit (NuGEN Technologies) prior to analysis. Integrity of the starting RNA was verified by Bio-Analyzer gel electrophoresis analysis (RIN scores ranged from 7.1 to 9.8). Oligonucleotide primer sequences used for the detection of *ITGA6*, *DISL3* (rt-PCR) and *POU5F1*, *EPN2*, *HeatR6* (qPCR) transcripts are listed in **S1 Table**. Primer oligonucleotides used in this study were designed to be intron-spanning to avoid amplification of genomic DNA during the reaction. RT-PCR reactions were set up using 1x PCR buffer containing 0.5U Taq polymerase (both Roche), 0.5 μM of forward and reverse primer and 2mM dNTPs in a final volume of 25 μl.

Quantitative PCR reactions were carried out in triplicate 10 μl reactions and reactions were set up using 2.5 ng amplified cDNA, 2x Roche cyber green mix (SY Green Master mix, Roche), 0.2 μM of forward and reverse primer in a final volume 10 μl. Fluorescence per cycle was measured on a LightCycler480 PCR machine (Roche). Specificity of the PCR reactions was ascertained through detection of single bands on agarose gel of the amplified products. We utilized the LinReg method [52] to calculate the starting concentration (N0 value) of *POU5F1*, *EPN2* and *HeatR6* transcripts in a given sample based on the fluorescence level per cycle number. Products of PCR were separated on a 3% agarose TBE gel containing ethidium bromide and visualized on a Gel Doc XR system (Bio-Rad).

## Results

**Fig 1:**
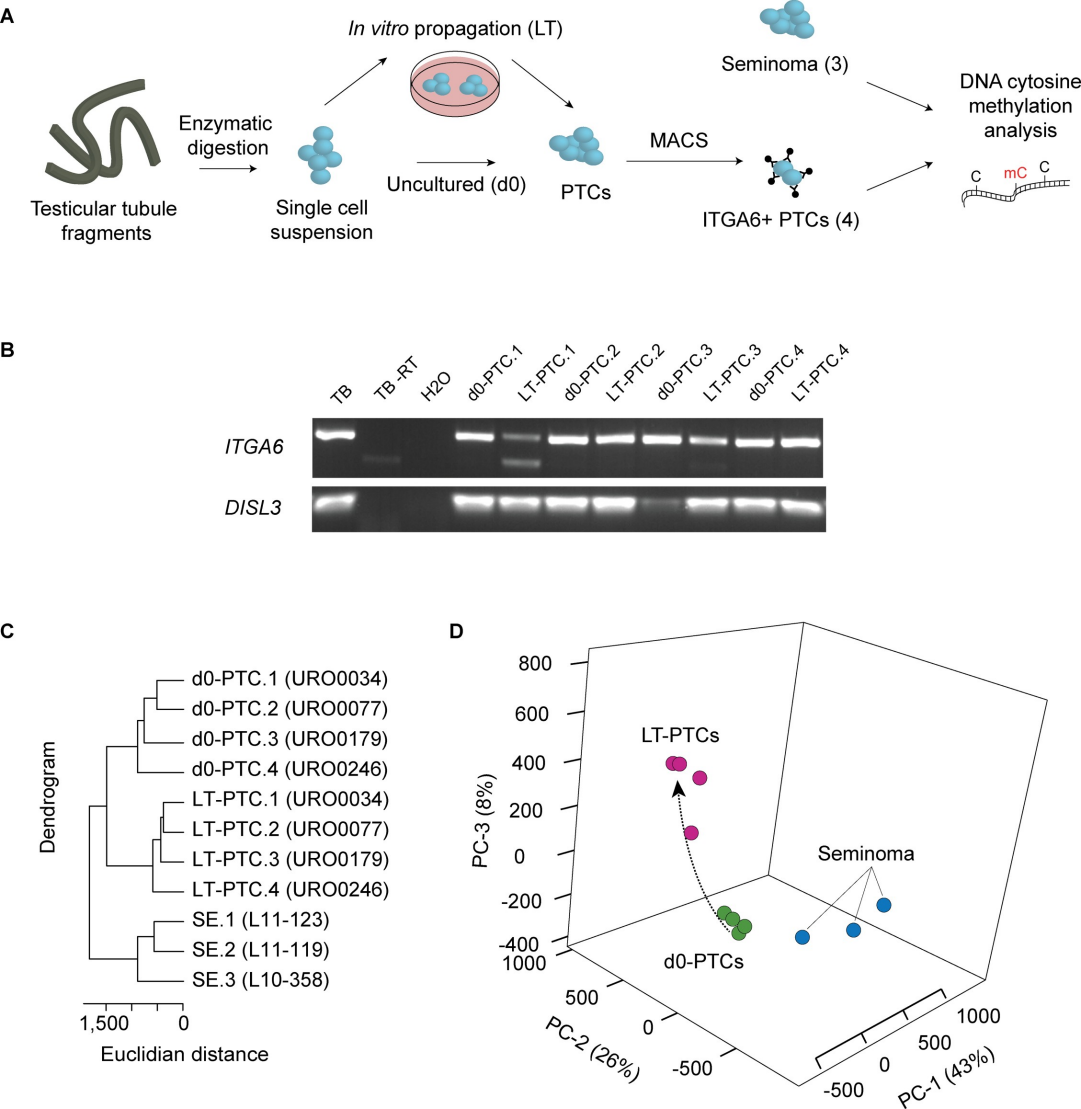
Generation of genome-scale DNA methylation profiles in primary cultured and sorted ITGA6+ testicular cells (PTC) and seminomas tumors (SE). In total, four PTC cultures and three primary seminoma samples were analyzed using the Illumina HumanMethylation 450k microarray platform. (A) Testicular tubule fragments were dissociated using enzymatic digestions and the resulting single cell suspension was either directly sorted for ITGA6+ cells (d0, day 0) or first cultured for 50–54 days (LT, long-term) and then sorted. Both d0-PTC/LT-PTC sorted fractions and SE were subjected to DNA cytosine methylation analysis. (B) Expression of *ITGA6* and *DISL3* (reference gene) mRNA transcripts in d0-PTCs and LT-PTCs. Total testicular tissue biopsy (TB) was used as a positive control, a cDNA synthesis using demineralized H2O as input was used as a negative control. (C) Unsupervised hierarchical clustering revealed unique DNA methylation landscapes in LT-PTCs and seminomas as compared to d0-PTCs with a clear segregation of PTCs and seminomas into distinct groups. (D) 3D principal component analysis (PCA) shows that the observed variance in DNA methylation levels in LT-PTCs and SE samples as compared to d0-PTCs occurs at different genomic regions. d0-PTCs samples are displayed in green, LT-PTCs in pink and SE in blue.

To study the possible transformation of normal human testicular cells into malignant cells during prolonged cell culture, we generated genome-scale 5-mC profiles of ITGA6+ enriched primary testicular cells (PTC, n = 4) both before culture (d0-PTC) and after long-term culture (LT-PTC), as well as three primary seminoma lesions (SE, n = 3) (**Fig 1A**). The general consensus in the literature is that spermatogonial stem cells (SSC) represent the fraction of male germ cells that are able to self-renew and differentiate into sperm via the process of spermatogenesis (reviewed in [53]). A distinction is made between SSCs and spermatogonia, the latter of which is an umbrella term for sperm cell precursors including both veritable SSCs as well as more differentiated premeiotic germ cell types that do not possess stem cell potential. We have previously demonstrated that our culture method, either with or without selection of ITGA6+ cells, yields a population of cells that have the ability to migrate to the basal membrane of the seminiferous tubules after xenotransplantation to mouse recipients, as evidenced by positive immunofluorescent staining of COT-1 DNA sequences (a sequence exclusively present in human DNA) in the transplanted tissues [40, 43, 54]. Although human SSCs cannot differentiate in the mouse testis microenvironment due to the large phylogenetic difference between mice and humans [17], these findings form the golden standard to evaluate the presence of spermatogonial stem cells in a transplanted population of cells and confirm that the human ITGA6+ testicular population contains SSCs.

As expected, d0-PTCs and LT-PTCs displayed continued expression of the *ITGA6* mRNA transcript during culture (**Fig 1B**). Unsupervised hierarchical clustering revealed clear segregation of the d0-PTC, LT-PTC and seminoma samples into three separate groups, highlighting a distinct DNA methylation profile in seminomas as compared to PTCs both before and after culturing (**Fig 1C and 1D**).

**Fig 2:**
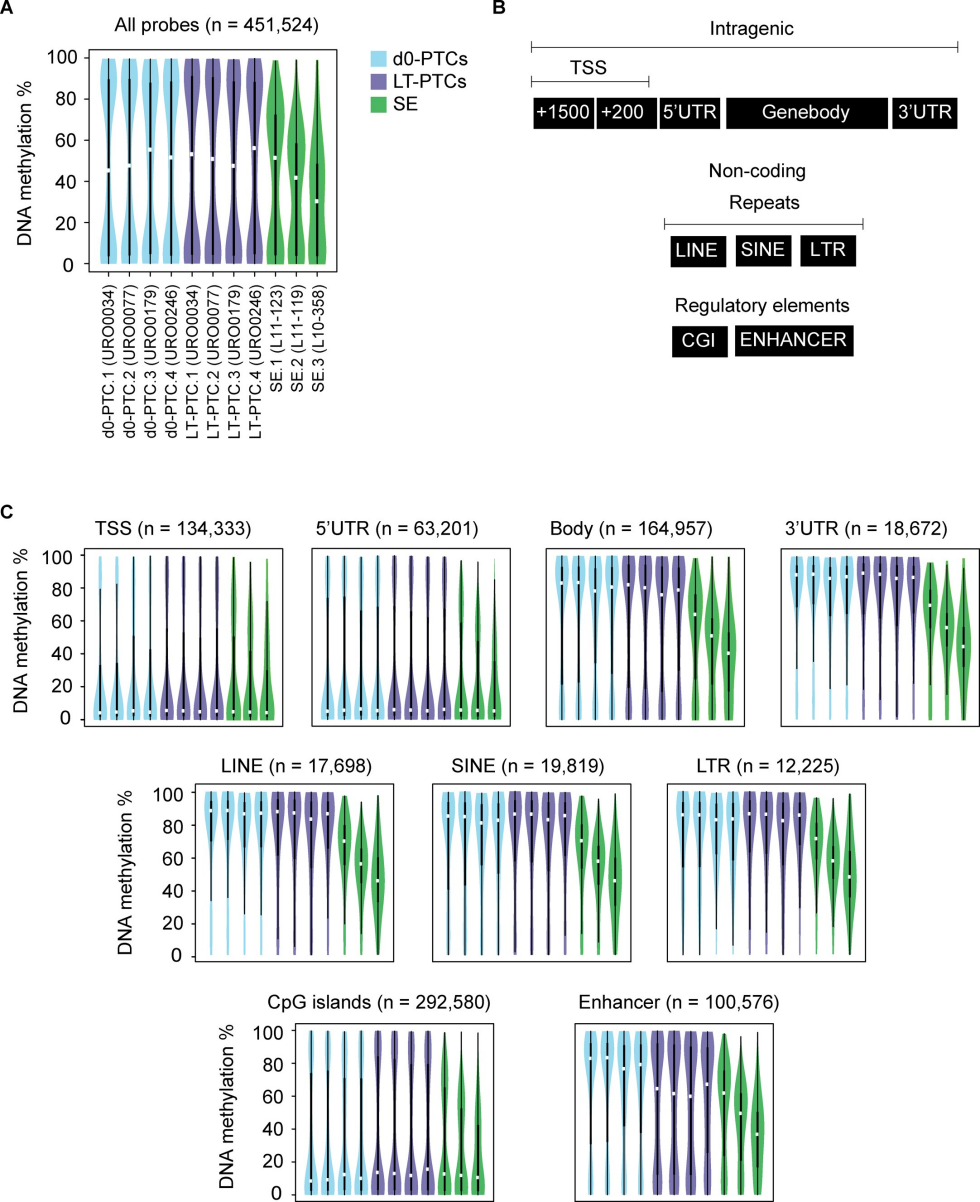
SE samples display a generally hypomethylated DNA methylation profile as compared to d0-PTCs and LT-PTCs. (A) DNA methylation is deposited in a bipolar fashion in d0-PTCs and LT-PTCS, with most probes displaying a DNA methylation of either 0–10% or 90–100%, while a clear global hypomethylation is observed in SE. (B) Probe DNA methylation data was divided into nine genomic classes according to the genomic annotation of probes to the hg38 human genome reference build. A distinction was made between intragenic sequences (+1500bp of transcription start site (TSS), +200bp of TSS, 5' untranslated region (UTR), first exon, rest of genebody and 3'UTR) and non-coding sequences (long interspersed elements (LINE), short interspersed elements (SINE), long terminal repeats (LTR), CpG islands (CGI) and enhancer regions). (C) Distribution of DNA methylation over the nine genomic classes in d0-PTCs, LT-PTCS and SE.

When analyzing the DNA methylation distribution over all included CpG probes (n = 451,524 CpGs), we found that seminomas display a distinct globally hypomethylated profile compared to cultured and uncultured PTCs (**Fig 2A**). Global genomic hypomethylation is a well described feature of tumor cells and seminomas in particular [28, 55–57], contrasting with normal cells which have a bimodal distribution of either fully methylated CpGs (100% methylation) or unmethylated CpGs (0% methylation). The extent of hypomethylation in seminomas correlated negatively with the lymphocyte infiltration score, i.e. the tumor possessing the lowest degree of lymphocyte infiltration (L10-358, 37% lymphocyte infiltration) had the most severely hypomethylated profile. This corroborates the findings of Shen et al., who recently studied 137 TGCTs and described the existence of multiple subtypes of seminoma lesions that are distinguishable in terms of lymphocyte infiltration, genetic mutations (*KIT* locus) and the extent of global DNA hypomethylation [58].

To further study the extent of DNA hypomethylation in seminomas compared to uncultured and cultured PTCs, we next analyzed CpG methylation levels at nine defined genomic classes; transcription start sites (TSS), 5’-UTR, gene bodies, 3’-UTR, long and short interspersed elements (LINE and SINE, respectively), long terminal repeats (LTR), CpG islands and enhancer regions (**Fig 2B**). In line with the observed global DNA hypomethylation, six of these nine classes were hypomethylated in seminomas as compared to d0-PTCs and LT-PTCs, namely gene bodies, 3’UTR, LINE, SINE, LTR and enhancer regions (**Fig 2C**). Interestingly, DNA methylation levels were found to be normally distributed in all groups at the promoter regions (TSS and 5’UTR) and CpG islands. Together, these data suggest that LT-PTCs do not acquire an overall deregulated epigenetic status as is shown in seminoma with a concurrent general loss of DNA methylation, as well as possible decreased genetic stability due to demethylation at retrotransposon sequences.

In order to determine which genomic sequences were differentially methylated between groups, we selected CpG-dense regions represented on the array and tested each region for statistically significant differences in methylation (differentially methylated region, DMR). CpG-dense regions were defined as genomic sequences where a minimum of 4 CpG probes are located within 200bp of each other, resulting in 28,901 regions of interest to be statistically tested. Adhering to significance cut-offs of an adjusted Benjamini-Hochberg corrected p-value ≤ 0.01 and a minimum median methylation difference of 25% between groups, we identified a total of 353, 486 and 550 DMRs between d0-PTCs/LT-PTCs, LT-PTCs/SE and d0-PTCs/SE, respectively.

**Fig 3:**
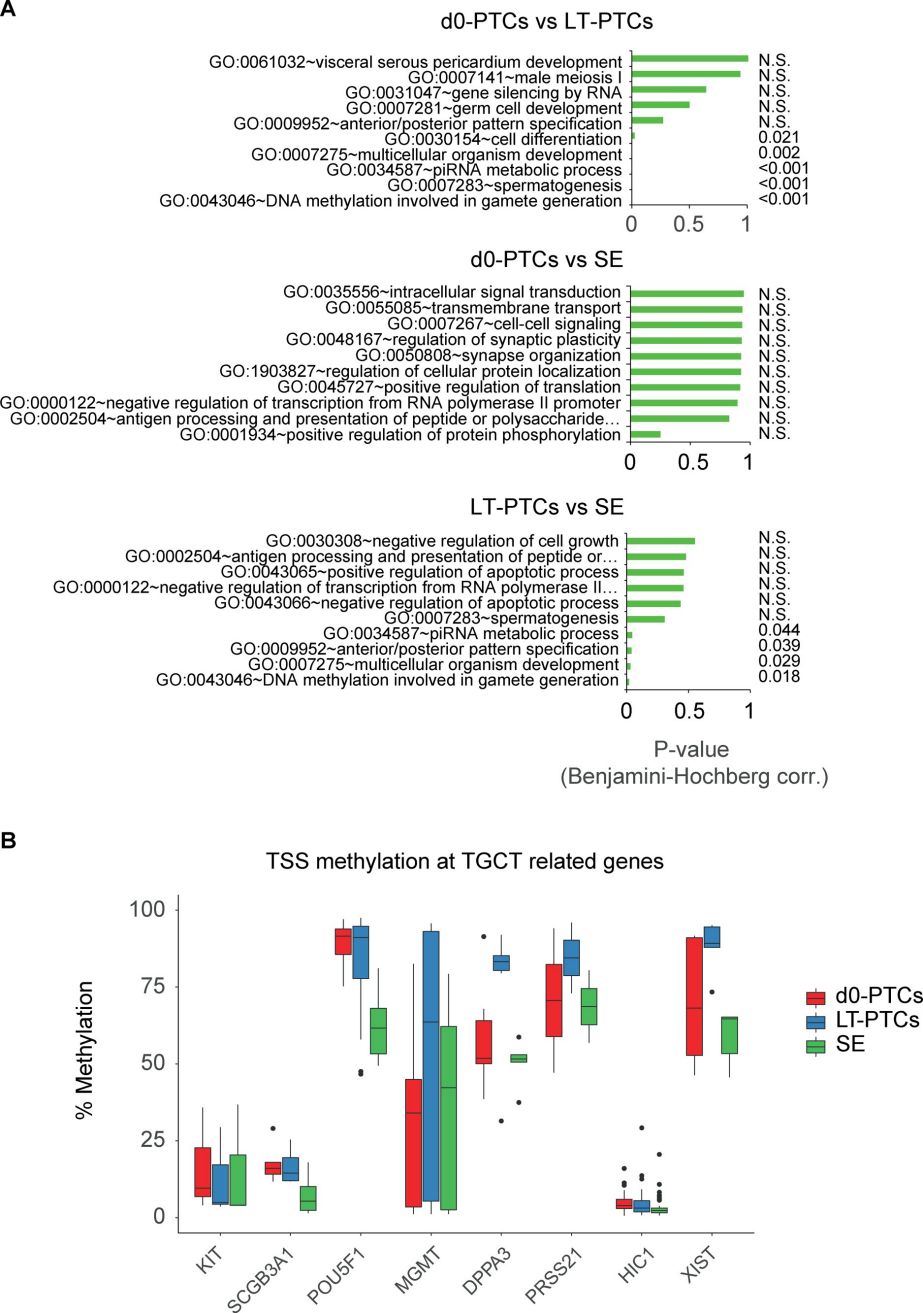
Detection of differentially methylated regions in d0-PTCs, LT-PTCs and seminomas. (A) Top 10 most enriched GO-terms (bottom to top) of DMRs between d0-PTC/LT-PTC, d0-PTC/seminoma and LT-PTC/seminoma. N.S. = not significant. (B) Gene-specific DNA methylation levels at tumor suppressor genes (*SCGB3A1*, *MGMT*, *PRSS21*, *HIC1*), testis-specific genes (*KIT*, *DPPA3*), *XIST* and the pluripotency marker *POU5F1*.

GO-enrichment analysis using the online DAVID tool [59] revealed enrichment of biological processes between d0-PTCs and LT-PTCs related to spermatogenesis and meiosis (**Fig 3A**). The top enriched GO-term “DNA methylation involved in gamete generation” (GO:0043046) contains several key regulators of male spermatogenesis; *PICK1*, *MAEL*, *ASZ1*, *PIWIL2*, *MOV10L1*, *DDX4* and *TDRD1* were differentially methylated in LT-PTCs as compared to d0-PTCs. All DMRs in this GO-term displayed increased methylation in LT-PTCs (increase of 48% ± 5.7% as compared to d0-PTCs) with the exception of *MOV10L*, which displayed decreased methylation (54%). In the LT-PTC versus seminoma comparison we found 4 enriched GO-terms that share a high degree of similarity with the terms found in the d0-PTC versus LT-PTC comparison, suggesting that long-term culture results in DNA methylation differences in LT-PTCs as compared to both d0-PTCs and seminomas, seemingly unrelated to malignant progression. There were no GO-terms between d0-PTCs and seminoma samples that reached statistical significance, possibly due to statistical power being too low or p-value cut-off levels.

Seminomas are known to show selective hypermethylation at several tumor suppressors, such as *MGMT*, *SCGB3A1*, *RASSF1A*, *HIC1*, and *PRSS21* [60]. In addition, seminomas can display genetic mutations in the *KIT* gene and they express the pluripotency marker *POU5F1*, which is not detectable in normal testis [61]. Analysis of DNA methylation levels at the TSS in specific tumor-related genes revealed a decreased promoter methylation level for *POU5F1* in seminomas (approximately 35% reduction in DNA methylation) as compared to both d0-PTCs and LT-PTCs, while we did not detect significant differential methylation in *SCGB3A1*, *PRSS21* or *HIC1* (**Fig 3B**). The *DPPA3* and *XIST* genes were hypermethylated in LT-PTCs as compared to d0-PTCs and seminomas, *MGMT* displayed a variable DNA methylation pattern between and within groups and the *KIT* promoter was stably hypomethylated in all groups including seminomas.

**Fig 4:**
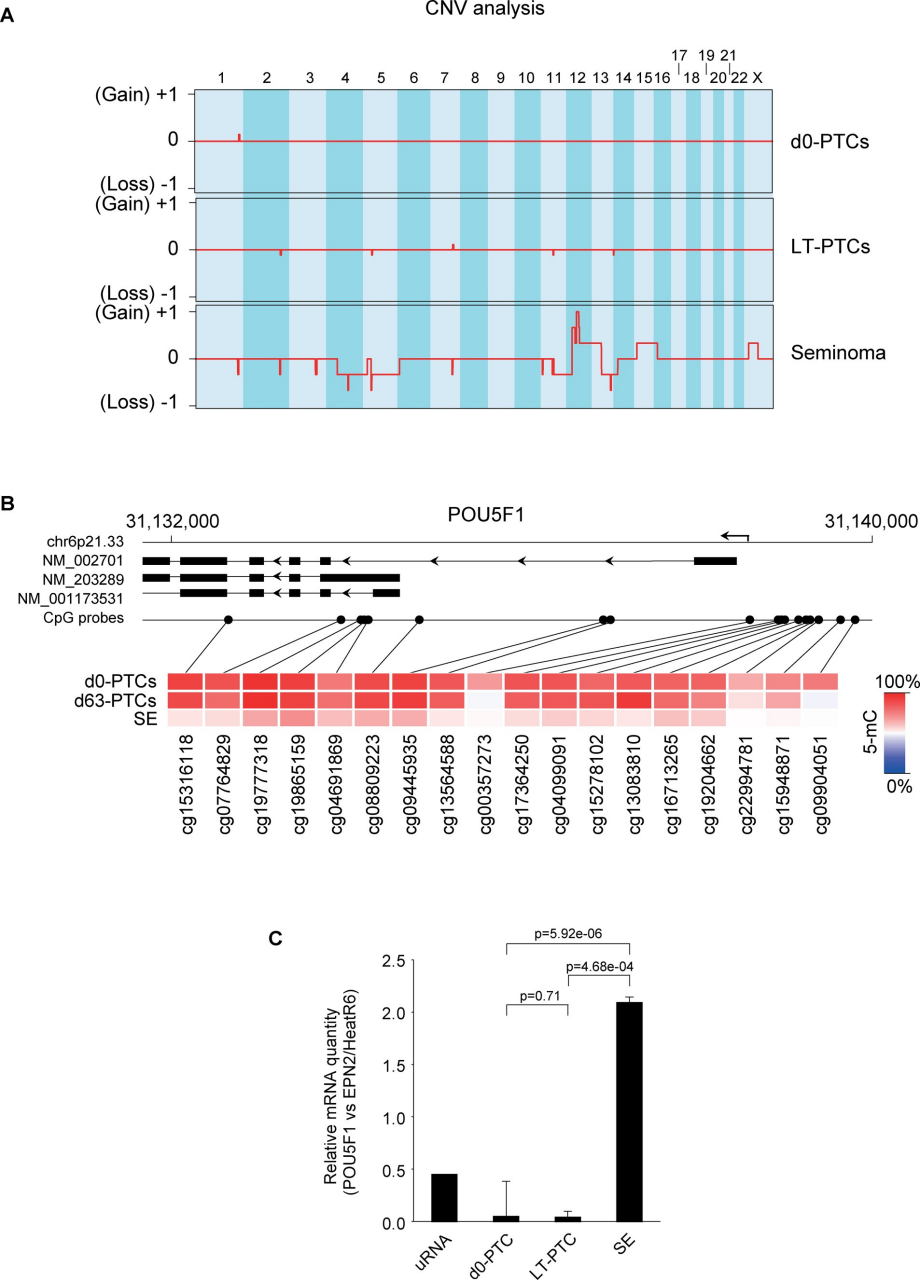
Copy number variation analysis reveals chromosomal aberrations in seminomas. Probe fluorescence data was used to calculate average intensity in 8,949 chromosomal bins covering all autosomal chromosomes (1–22) and the X chromosome as aggregates of each sample group (see Methods). Average increases in probe fluorescence are designated as 'Gains' and average decreases as 'Losses' with the y-axis describing the extent of 2 fold amplification or reduction (for example, a gain of +1 signifies a two-fold increase in copy number within that particular bin). (B) The *POU5F1* locus on chromosome 6p21.33 (also known as *OCT3/4*) is an oncogene that is activated in most germ cell cancers. SE samples displayed hypomethylation across the 18 CpG probes localized to this locus. Both d0-PTCs and LT-PTCs show a hypermethylated state of these CpG sites. (C) *POU5F1* mRNA expression normalized to the expression of two reference genes (*EPN2* and *HeatR6*) in d0-PTCs, LT-PTCs and seminoma samples as measured by quantitative fluorescence PCR. P-values were calculated using a two-sided student’s t-test. Universal RNA (uRNA) was used as a positive control.

In addition to loss of both global and specific DNA hypermethylation patterning, seminomas are known to acquire structural chromosomal aberrations such as extra p-arms of chromosome 12 (preferentially as isochromosome i(12p)) during progression to invasive growth [62]. Other non-recurrent chromosomal aberrations in seminomas include gains of chromosomes 7, 8, 12p, 21 and X, and loss of chromosomes 1p, 11, 13 and 18 with varying occurrence in individual tumors [63]. To determine whether chromosomal stability in PTC cultures is maintained, we compared CNVs in uncultured and cultured PTCs with that of the seminomas samples using a previously published approach to estimate CNVs from DNA methylation array data [51]. LT-PTCs displayed some small CNVs (chromosomes 2, 5, 7, 11 and 14) that did not reach the significance threshold, corresponding to heterogeneity within individual PTC cultures. Indeed, CNVs occurring in d0-PTCs and LT-PTCs groups were located to different chromosomal regions in each individual culture, suggesting the absence of persistent CNV ‘hotspots’ (**S1 File**). The seminomas samples showed large-scale losses and gains on several chromosomes including the staple i(12p) duplication, as well as gains of chromosome 15, 16 and X and losses on chromosomes 4, 5, 11 and 13 (**Fig 4A**).

Finally, we investigated the pluripotency marker *POU5F1* in more detail due to its importance in TGCT diagnostics. The *POU5F1* locus is covered by 18 CpG sites on the array platform that collectively displayed a hypomethylated state in seminomas as compared to d0-PTCs and LT-PTCs, including the genomic region directly upstream of the transcription start site (**Fig 4B**). To confirm the activated gene expression of *POU5F1* in seminomas we performed gene expression analysis of the *POU5F1* isoform 1 transcript (NM_002701) by qPCR and found high expression levels in seminomas while it was nearly undetectable in PTCs both before and after culturing (**Fig 4C**).

## Discussion

We here present evidence that *in vitro* propagated and ITGA6+ enriched primary human testicular cells display a normal global DNA methylation profile. Compared to seminoma, the uncultured and cultured ITGA6+ PTCs do not harbor large-scale CNVs nor show signs of oncogene or retrotransposon activation in terms of regional DNA demethylation. This suggests that, in contrast to the hypomethylated profile and copy number variants commonly observed in GCNIS-derived lesions, including seminomas [64–67], primary testicular ITGA6+ cells remain genetically stable during prolonged cell culture. In contrast, we did observe significant differences in DNA methylation between cultured and uncultured ITGA6+ cells, which did not resemble the epigenetic hallmarks of malignant progression but is potentially relevant for their prospective use in clinical transplantation procedures.

The absence of large-scale chromosomal duplications or losses is in line with an earlier publication where long-term cultured ITGA6+ testicular cells (over 50 days in culture) were screened using single-cell CGH arrays and were reported to be genetically stable (68), which we were able to confirm here. In that same study, epigenetic alterations in cultured ITGA6+ PTCs were reported in a panel of five imprinted loci (*H19*, *H19-DMR*, *MEG3*, *KCNQ1OT1* and *PEG3*) as compared to uncultured ITGA6+ PTCs and spermatozoa. The paternally imprinted *H19*, *H19-DMR* and *MEG3* loci were reported to show demethylation of 28%-11%, 68%-43% and 26%-18%, respectively, while the maternally imprinted *KCNQ1OT1* (13%-50%) and *PEG3* (30%-38%) loci were hypermethylated during culture.

The data presented here also support previous efforts (totaling 30–40 recipient mice) where uncultured and cultured human testicular cells were transferred to mouse testes using xenotransplantation and during which tumor formation was never observed or reported after careful histological analyses of the transplanted tissues [17, 43, 54, 68]. Consistent with these data, no increases in tumor incidence or lowered life expectancy after long-term follow up (18 months of age) were reported in recipient mice after transplantation of allogenic *in vitro* propagated SSCs [69]. It should be noted that the human testicular cell culture system used in this study differs from the mouse culture system in several aspects, including but not limited to the use of feeder cells. More specifically, while mouse spermatogonial cell cultures are established by culturing enriched SSC fractions on a monolayer of STO or MEF feeder cells treated with mitomycin [13, 70, 71], the human system utilizes untreated testicular somatic cells originating from the donor testis tissue itself as outgrowing feeder cells. The manner in which germ cells are established and maintained *in vitro* is vital to the stability and identity of the cultured germ cell fraction, and as such the results from mouse SSCT experiments do not necessarily reflect the human situation. More research is needed in the characterization of cultured human SSCs before and after (xeno)-transplantation.

Microarray analyses are inherently limited by the resolution of the design of the array and might not be suitable for the detection of low-grade cell mosaics, such as trace amounts of tumor cells in a large population of normal cells. In the CNV analysis, we adhered to the detection limits as suggested by Sturm et al. [51] which does not detect chromosomal copy number aberrations present in under 10% of cells. Similarly, because of the dynamic nature of DNA methylation patterning we screened our samples for differentially methylated sites that displayed a significant increase or decrease in DNA methylation between samples (25% or more) in order to maximally reduce technical noise and identify sites that are plausibly biologically relevant. In contrast to seminomas, the *POU5F1* transcript was virtually undetectable by qPCR in PTCs after culture, which would argue against the presence of even a small number of tumorigenic cells, although these data are not enough to rule out the presence of trace amounts of malignant cells completely. Moreover, while the 450k DNA methylation array platform is designed to include the majority of RefSeq genes, imprinting control regions (ICR) are not well covered in the design of the 450k array (less than 300 CpG sites covering ICRs (25)), and due to the low number of CpGs and relatively small sample size in our study we did not investigate these regions here. Based on these considerations, we stress that we are not able to fully exclude the presence of trace numbers of malignant cells in the cultured primary ITGA6+ testicular cell population.

There has been discussion in literature regarding the stability of germ cell DNA methylation patterns and the role of somatic contamination in the analyzed cell fractions. A recent study in marmoset monkey revealed stable DNA methylation patterns of the *H19*, *MEST*, *DDX4* and *MAGEA4* genes in germ cells cultured up to 21 days [72]. Since we detected differential methylation of GO-terms related to germ cell development and in particular DNA methylation regulation of germ cell formation, there could be differences in the functionality or cellular composition of the cultured cell fractions between human and marmoset/rodent germ cell cultures. There is some evidence of the role of epigenetic factors in the regulation of sperm development and quality [73] and the potential for transgenerational inheritance of epigenetic germ line mutations. As such, we strongly feel that more studies are required that focus on transcriptomic and epigenetic measurements in human germ cell cultures, for example through the use of novel emerging single-cell sequencing approaches [74–76], to gain insight in the events that occur during *in vitro* culture of human testicular cells.

## Conclusions

In conclusion, we show that human primary cultured and sorted ITGA6+ testicular cells do not possess large-scale CNVs or epigenetic hallmarks found in malignant cells. Nevertheless, we strongly advise additional studies that focus on assessing the consequences of culture-induced epigenetic alterations on the functionality of *in vitro* propagated germ cells prior to clinical transplantation, focused on identifying detailed characteristics of human SSCs *in vitro* and after (xeno)-transplantation, as well as developing more sensitive methods to detect and remove trace malignant cells from human testicular cell cultures prior to transplantation.

## Supporting information

S1 Table Primers used in the reverse transcriptase and quantitative PCR experiments. (XLSX)

S1 File Visualization of copy number variations in individual d0-PTC, LT-PTC and seminoma samples. CNV analysis using the total sum of unmethylated and methylated signals of the probes located on the Illumina HumanMethylation 450k beadchip array platform in individual samples. Cutoff values for the designation of copy number variant type is displayed on the y-axis on the right side of the plots, colored dots represent CNV type: homozygous deletion in brown, hemizygous deletion in red, neutral in grey, duplication in green and high-copy gain in blue.
(PDF)

S1 Raw images Agarose gel blots of the reverse transcriptase (*ITGA6*, *DISL3*) and quantitative (*POU5F1*, *EPN2* and *HeatR6*) PCR products. (PDF)
