# Supplementary figures and images for: Comparing genome-scale DNA methylation and CNV marks between adult human cultured ITGA6+ testicular cells and seminomas to assess in vitro genomic stability
Source: PLoS One. 2020 Mar 16;15(3):e0230253. doi: 10.1371/journal.pone.0230253 (PMC7075560; doi:10.1371/journal.pone.0230253)

d0-PTC.1 (0034)

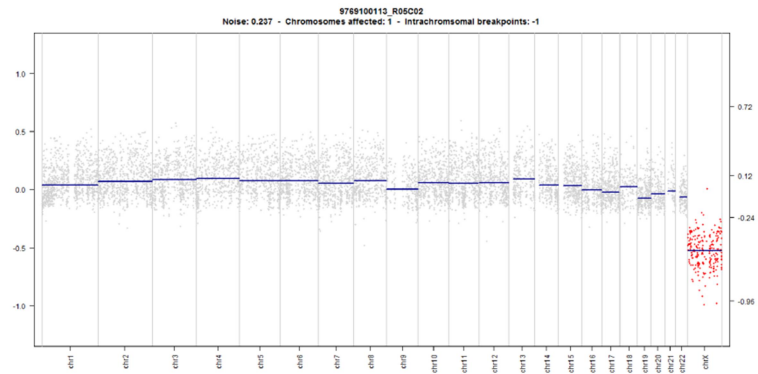

d0-PTC.2 (0077)

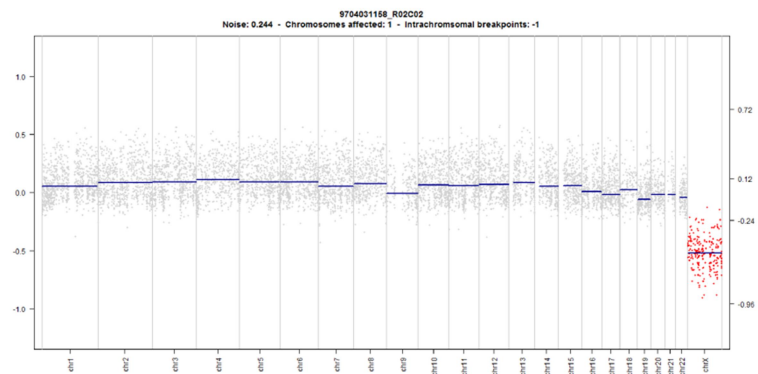

d0-PTC.3 (0179)

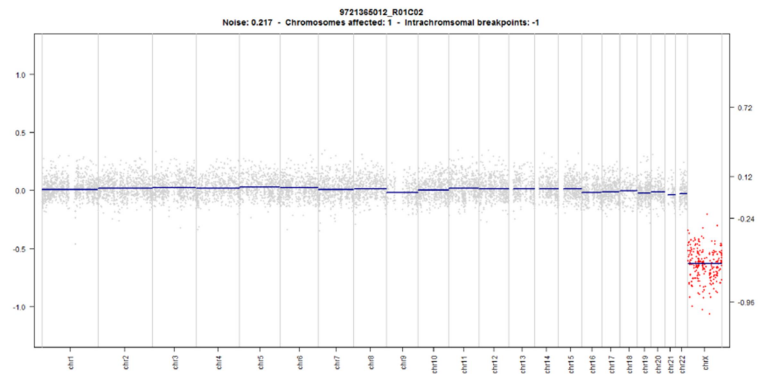

d0-PTC.4 (0246)

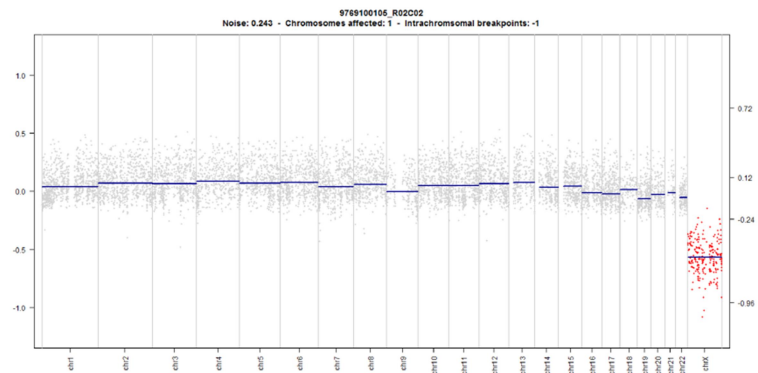

### LT-PTC.1 (0034)

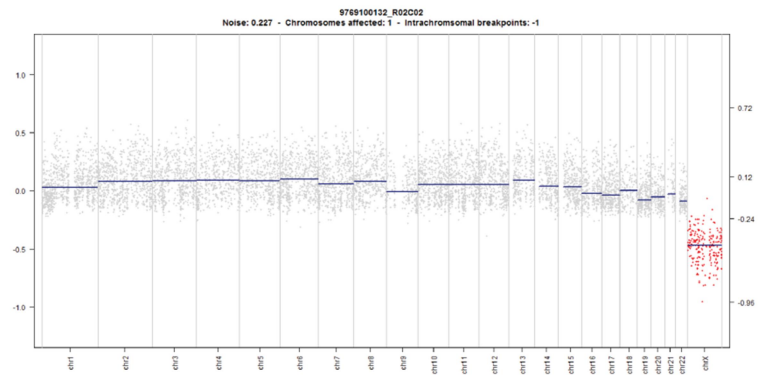

### LT-PTC.2 (0077)

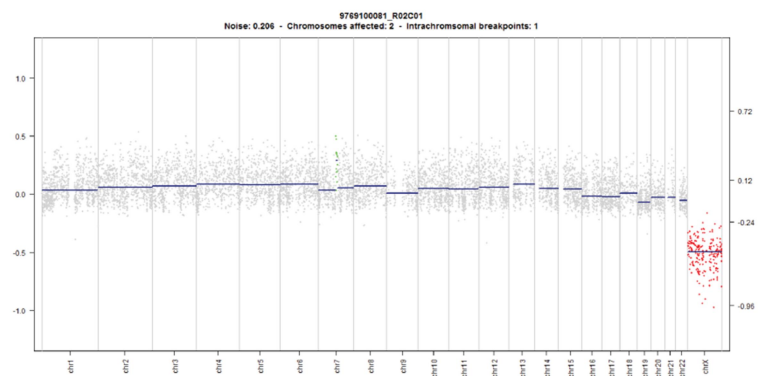

### LT-PTC.3 (0179)

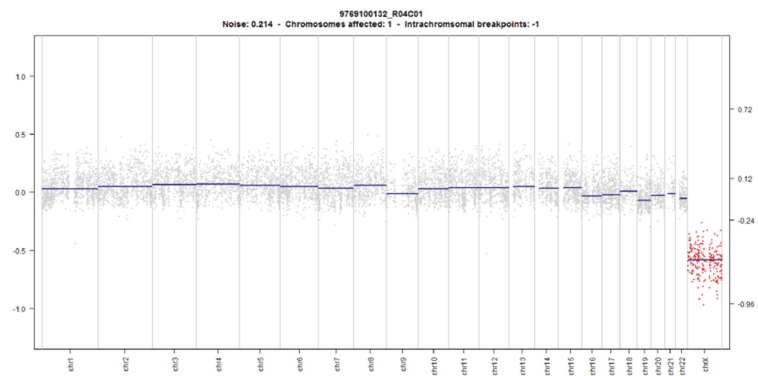

### LT-PTC.4 (0246)

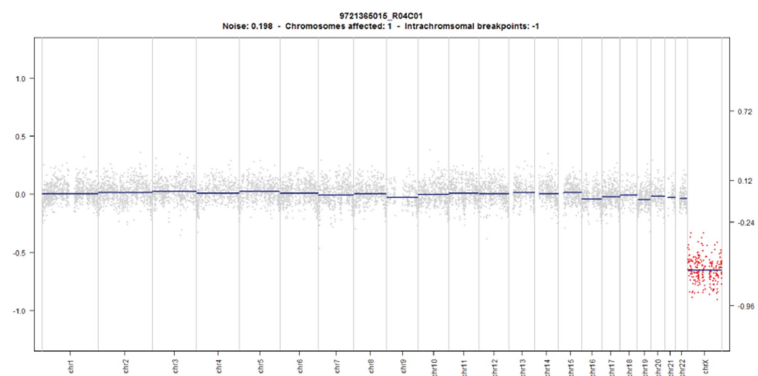

### SE.1 (358)

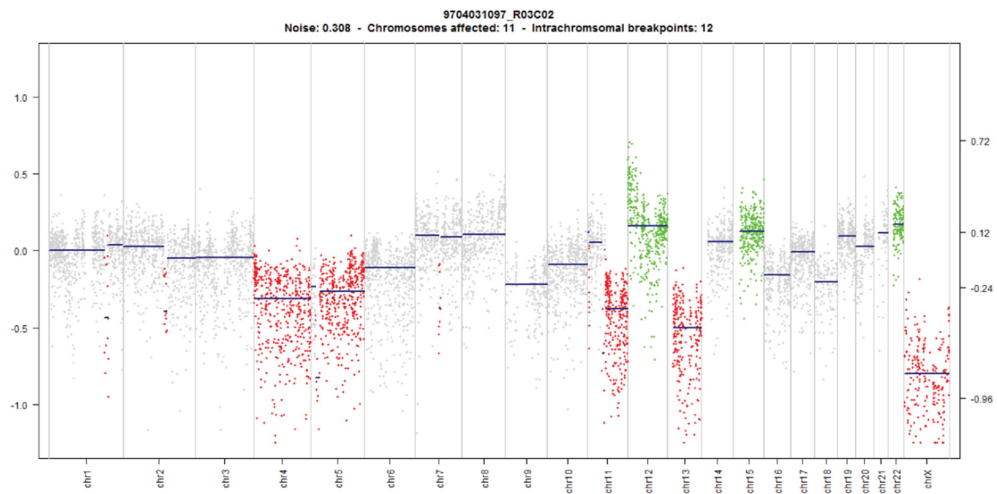

### SE.2 (119)

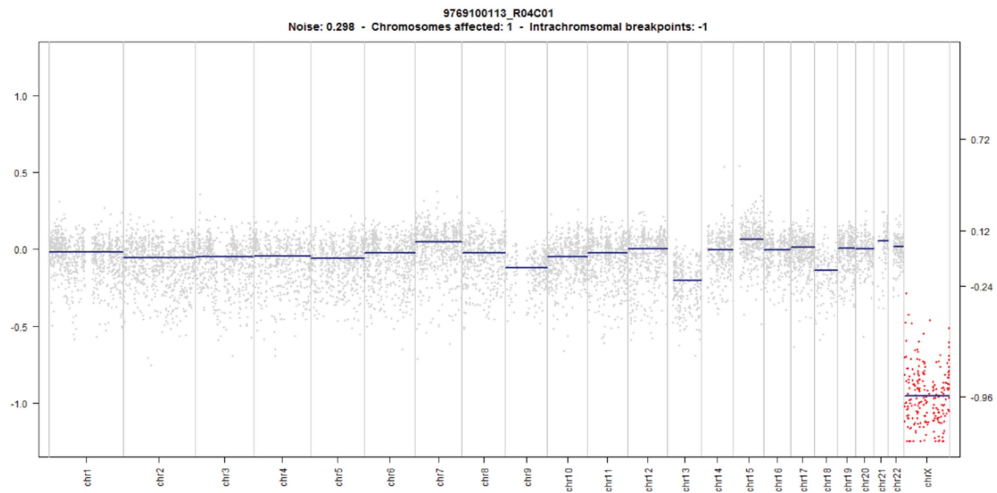

### SE.3 (123)

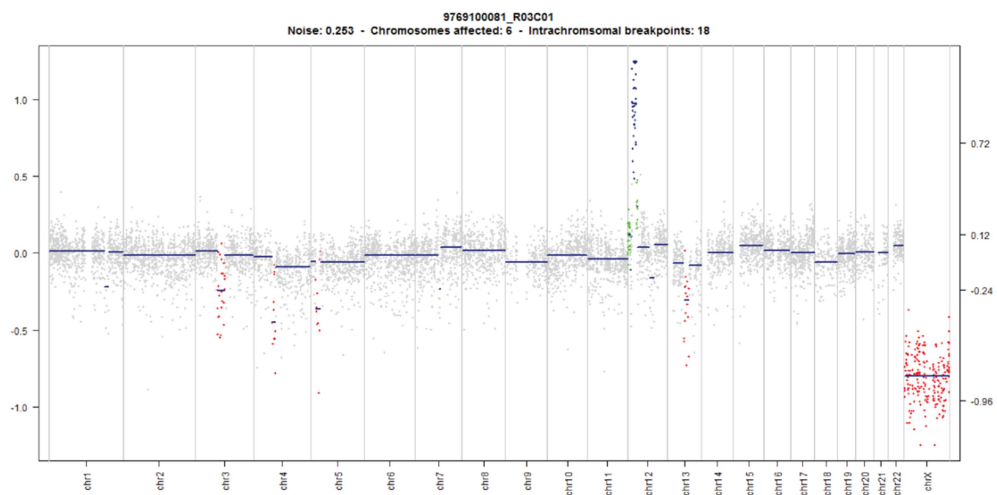

Supplement: S1 File — CNV analysis using the total sum of unmethylated and methylated signals of the probes located on the Illumina HumanMethylation 450k beadchip array platform in individual samples. Cutoff values for the designation of copy number variant type is displayed on the y-axis on the right side of the plots, colored dots represent CNV type: homozygous deletion in brown, hemizygous deletion in red, neutral in grey, duplication in green and high-copy gain in blue. (PDF) [file pone.0230253.s002.pdf]
