# Supplementary material for: Comparing genome-scale DNA methylation and CNV marks between adult human cultured ITGA6+ testicular cells and seminomas to assess in vitro genomic stability
Source: PLoS One. 2020 Mar 16;15(3):e0230253. doi: 10.1371/journal.pone.0230253 (PMC7075560; doi:10.1371/journal.pone.0230253)
Supplement: S1 Raw images — (PDF) [file pone.0230253.s003.pdf]

POU5F1

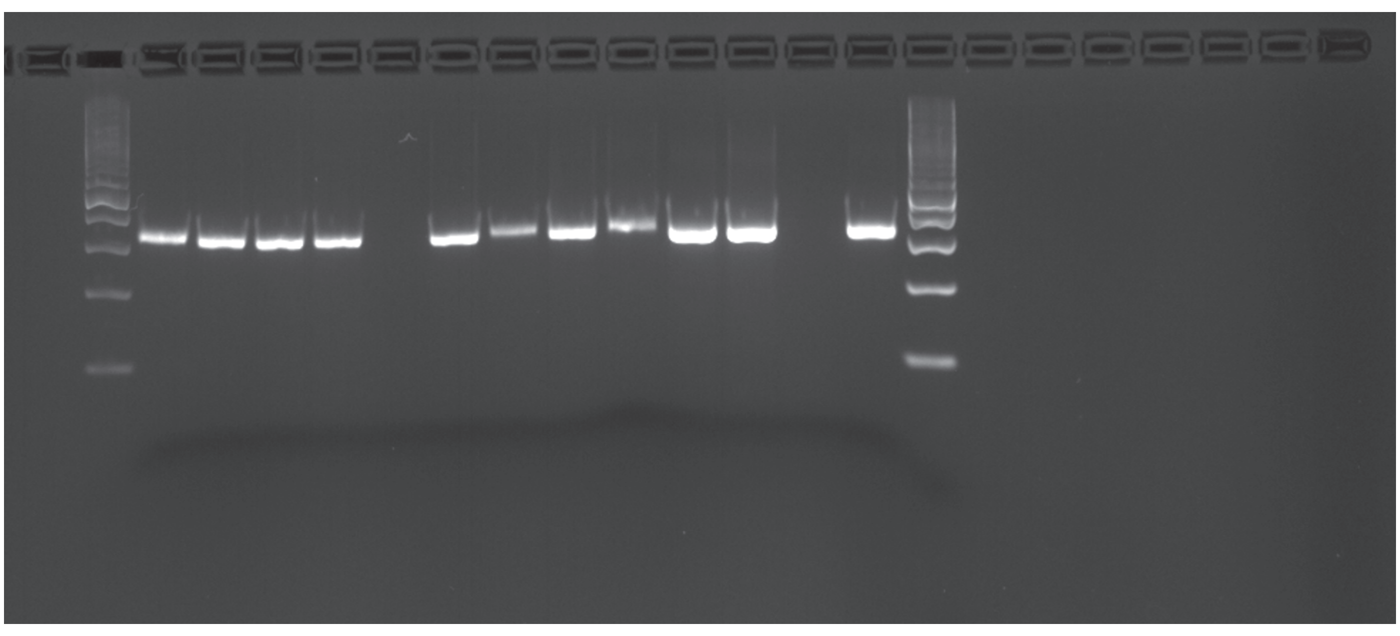

1. Marker
2. d0-PTC.1
3. d0-PTC.2
4. d0-PTC.3
5. d0-PTC.4
6. LT-PTC.1
7. LT-PTC.2
8. LT-PTC.3
9. LT-PTC.4
10. SE.L10-358
11. SE.L11-111
12. SE.L11.123
13. uRNA (pos ctrl)
14. H2O (neg ctrl)
15. Marker

EPN2

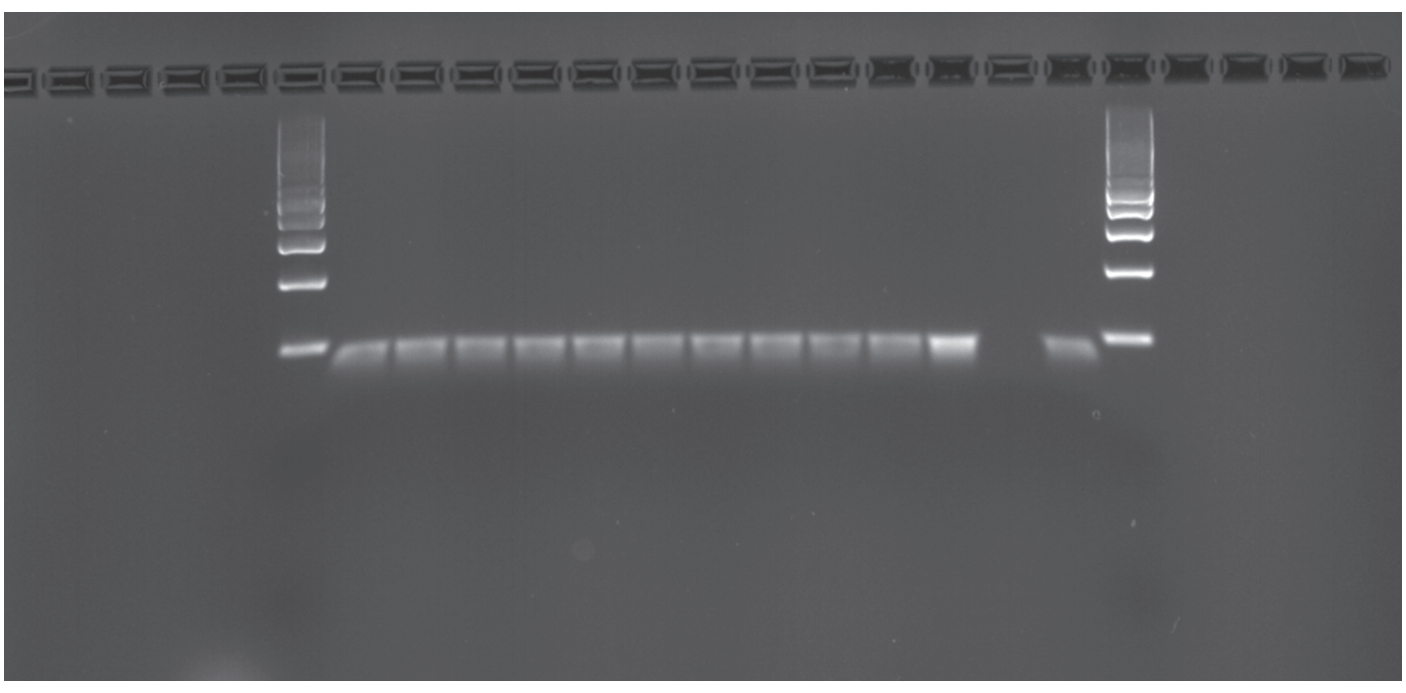

1. Marker
2. d0-PTC.1
3. d0-PTC.2
4. d0-PTC.3
5. d0-PTC.4
6. LT-PTC.1
7. LT-PTC.2
8. LT-PTC.3
9. LT-PTC.4
10. SE.L10-358
11. SE.L11-111
12. SE.L11.123
13. uRNA (pos ctrl)
14. H2O (neg ctrl)
15. Marker

HeatR6

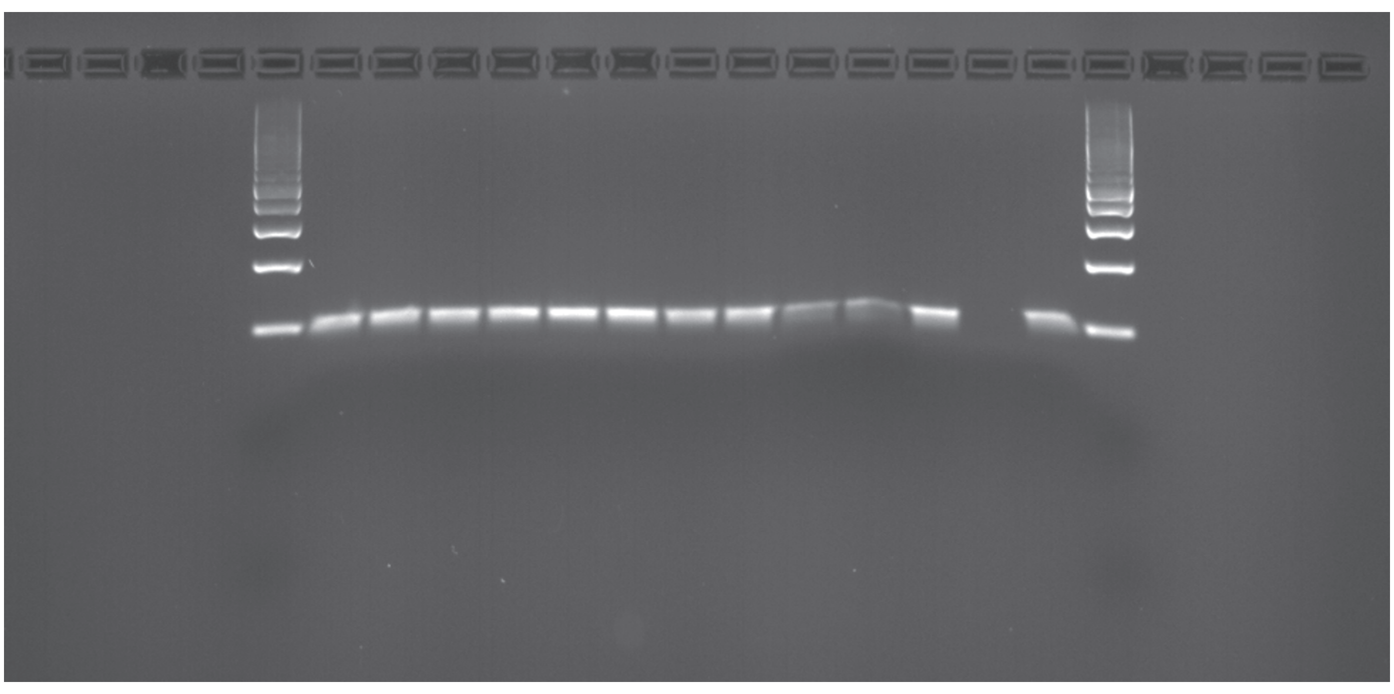

1. Marker
2. d0-PTC.1
3. d0-PTC.2
4. d0-PTC.3
5. d0-PTC.4
6. LT-PTC.1
7. LT-PTC.2
8. LT-PTC.3
9. LT-PTC.4
10. SE.L10-358
11. SE.L11-111
12. SE.L11.123
13. uRNA (pos ctrl)
14. H2O (neg ctrl)
15. Marker

ITGA6

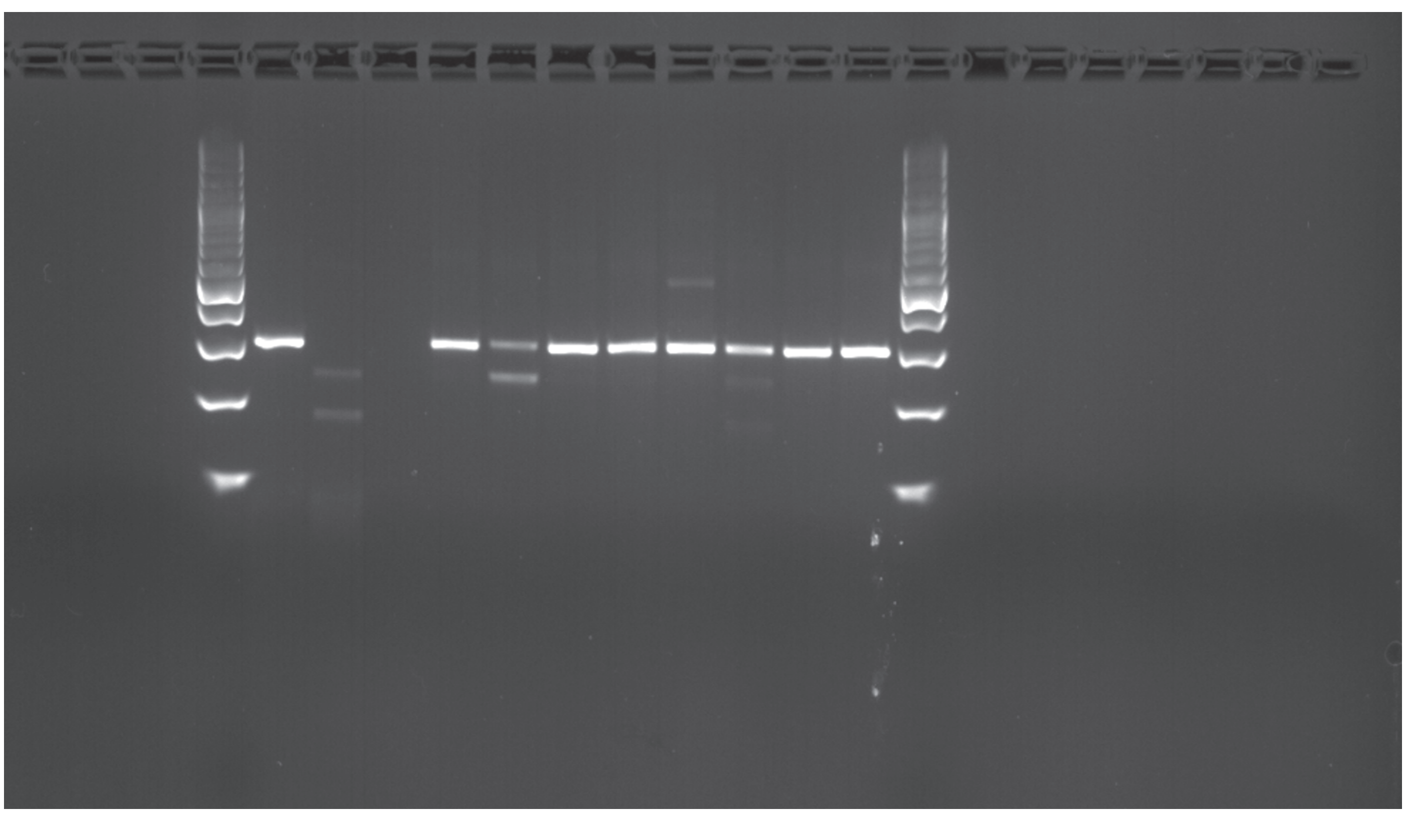

1. Marker
2. TB
3. TB -RT
4. H2O ctrl.
5. d0-PTC.1
6. LT-PTC.1
7. d0-PTC.2
8. LT-PTC.2
9. d0-PTC.3
10. LT-PTC.4
11. d0-PTC.4
12. LT-PTC.4
13. Marker

DISL3

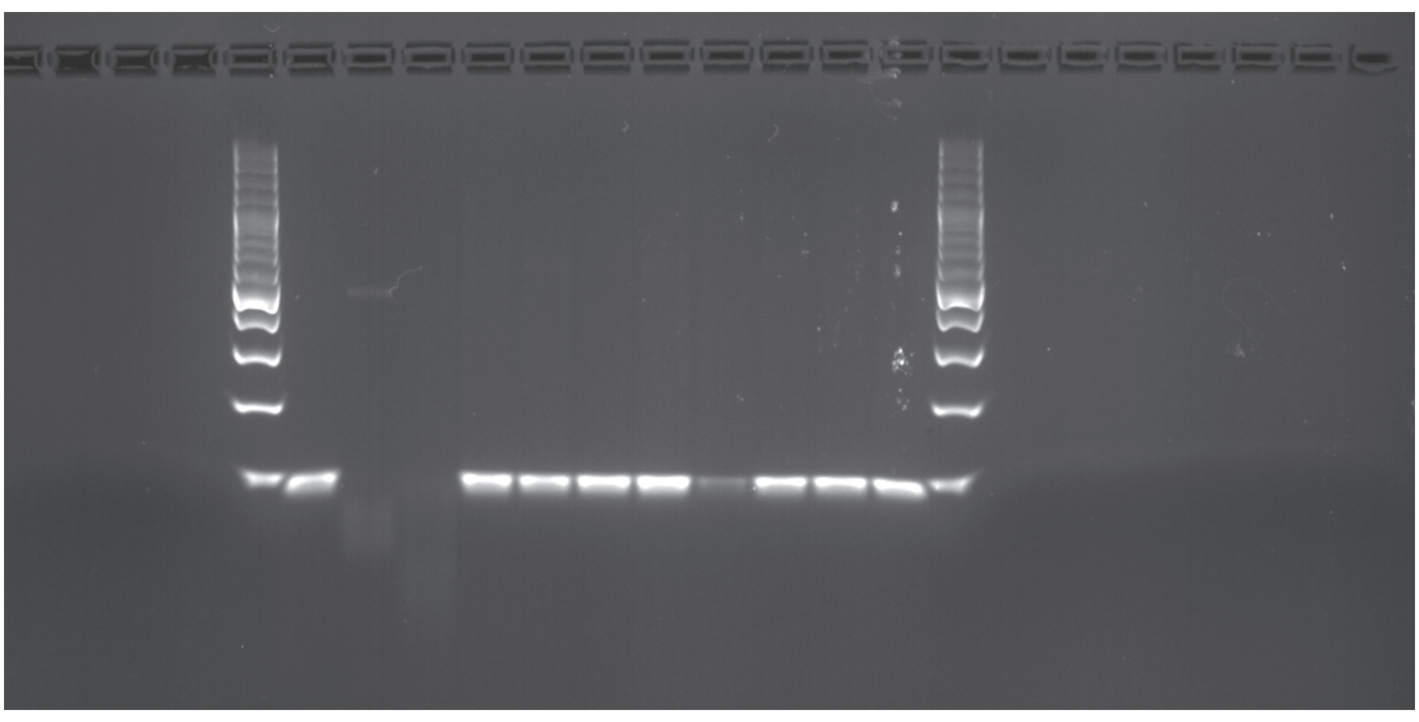

1. Marker
2. TB
3. TB -RT
4. H2O ctrl.
5. d0-PTC.1
6. LT-PTC.1
7. d0-PTC.2
8. LT-PTC.2
9. d0-PTC.3
10. LT-PTC.4
11. d0-PTC.4
12. LT-PTC.4
13. Marker
